# *grdA* on different plasmids and chromosomes of *Salmonella enterica*

**DOI:** 10.1128/aac.00669-25

**Published:** 2025-09-22

**Authors:** Zhiqiu Yin, Xiaoyan Tian, Zhanpeng Yu, Mujie Zhang, Baozhu Chen, Yanling Zhang, Liang Peng

**Affiliations:** 1 Department of Clinical Laboratory, Key Laboratory of Biological Targeting Diagnosis, Therapy and Rehabilitation of Guangdong Higher Education Institutes, The Fifth Affiliated Hospital, Guangzhou Medical University 26468, Guangzhou, Guangdong, China; 2 KingMed School of Laboratory Medicine, Guangzhou Medical University 26468, Guangzhou, Guangdong, China; University of Fribourg, Fribourg, Switzerland

**Keywords:** *grdA*, *Salmonella enterica*, genomics, plasmid, chromosome

## LETTER

A novel aminoglycoside resistance gene, *grdA*, was initially identified as a plasmid-borne gentamicin resistance determinant in *Salmonella enterica* (1). More recently, Hikal et al. demonstrated its role in conferring high-level resistance (MIC > 256 µg/mL) to plazomicin, a next-generation aminoglycoside recently approved by the U.S. Food and Drug Administration (FDA) for the treatment of multidrug-resistant (MDR) complicated urinary tract infections (2). While the initial characterization of *grdA* highlighted its location on the Col-type plasmid pZJ18, subsequent analyses have indicated its possible presence on different plasmids or chromosomes (1). Therefore, characterizing the genomic dynamics of *grdA* will enhance our understanding of the resistance dissemination and the clinically relevant risk assessment, particularly in the context of increasing plazomicin resistance. To address this, we performed a systematic genomic investigation of *grdA*-harboring *S. enterica* isolates, integrating comparative genomics, phylogenetics, and resistome profiling to unravel its diverse genomic locations, chromosomal and plasmid dynamics, and co-occurrence with antimicrobial resistance (AMR) genes.

To characterize bacterial genomes harboring *grdA* genes, we performed a search using the NCBI BLASTn (accessed on 9 April 2025) against the nucleotide collection (nr/nt) database. Nine complete genomes of *S. enterica* subsp. *enterica* containing *grdA* were identified, with strains classified into the serovars Bredeney (*n* = 3), Albany (*n* = 2), and Heidelberg (*n* = 4) (Table S1). To place these samples into a wider context, we also included other complete genome assemblies of three serovars (comprising 6 Bredeney, 13 Albany, and 56 Heidelberg genomes) from the NCBI GenBank database (Fig. S1). Gene annotation was performed uniformly via Prokka v1.14.5 (3). The resulting GFF files were analyzed using Panaroo v 1.3.4 with the default settings to determine the core gene alignment (4), which was used to construct the maximum-likelihood tree using IQ-TREE v2.2.5 with the general time reversible model and 1,000 bootstrap replicates (5). PlasmidFinder v.2.0 (Center for Genomic Epidemiology) was employed for screening plasmid replicons (6). AMR genes were detected using ResFinder v4.3.1 (7), with a cutoff at 80% nucleotide identity and 80% nucleotide coverage. The features of the genome and comparisons thereof were generated and visualized using the BLAST Ring Image Generator (8) and Easyfig 2.2.5 (9).

**Fig 1:**
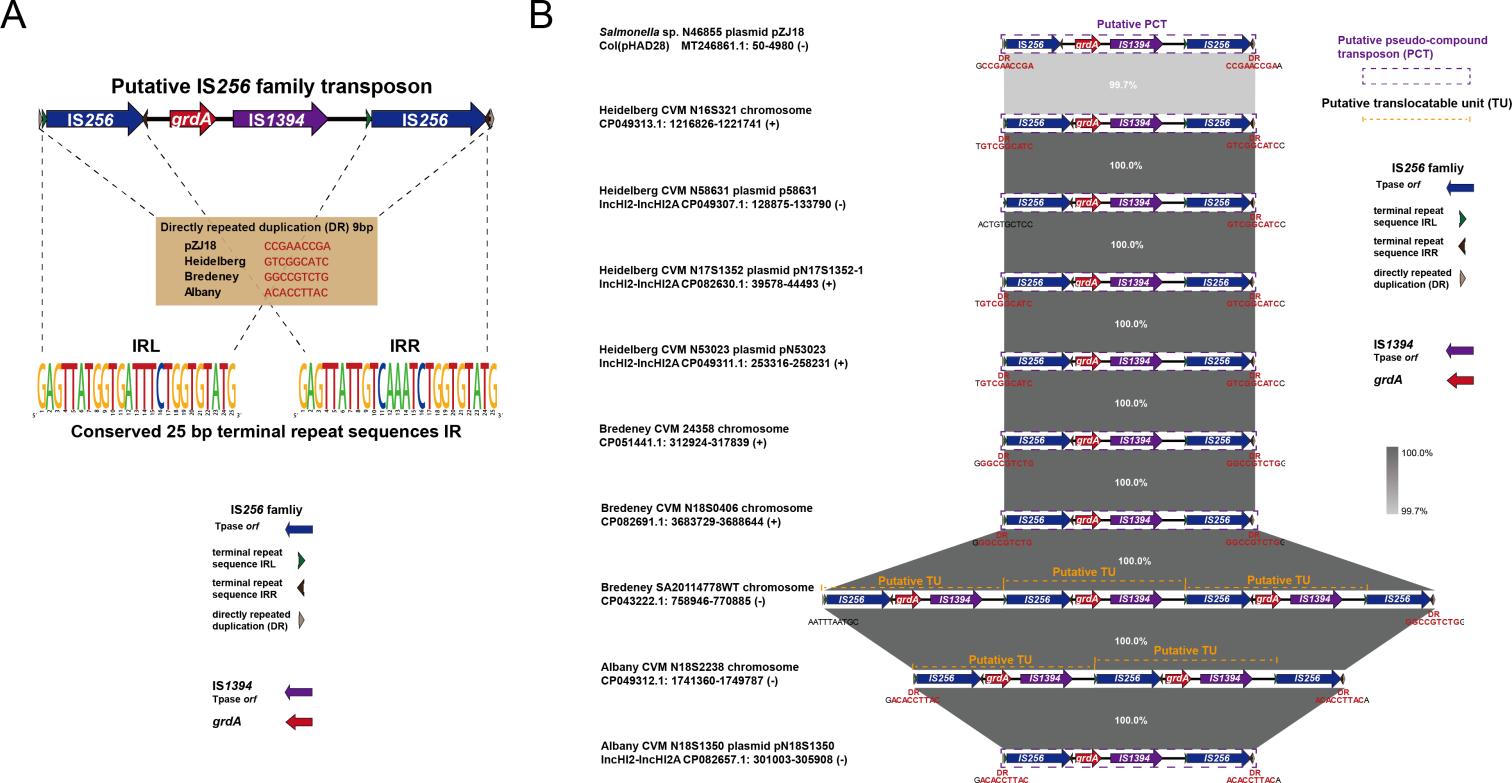
(**A**) The genetic organization of IS*256-grdA*-IS*1394*-IS*256*. (**B**) Linear comparison of IS*256-grdA*-IS*1394*-IS*256* in *S. enterica*. Genes are shown as arrows and indicated according to their putative functions. Dark blue indicates IS*256*; purple indicates IS*1394*; red represents *grdA*. Green, dark brown, and light gray triangular arrows represent IRL, IRR, and DR, respectively. Regions with a high degree of homology are indicated by gray shading, in conjunction with Easyfig BLASTn % nt identity.

All nine of these strains were isolated from turkey samples. Eight of them were part of the U.S. FDA surveillance project for the U.S. National Antimicrobial Resistance Monitoring System (NARMS) (10). Notably, while both these isolates and those of Kim et al. analyzed *grdA*-positive *Salmonella* isolates from U.S. retail turkey samples within the NARMS framework (1), the eight strains examined here represent distinct isolates from those originally reported by McDermott et al. (BioProject: PRJNA242614) (11) (Table S2). Furthermore, Hikal et al. identified *grdA* in 2.7% (592/22,276) of *S. enterica* isolates in the NCBI database (2). However, the nine strains analyzed herein represent the only complete genomes currently available that contain *grdA*, thus enabling detailed structural resolution of its genomic context. The remaining strain (SA20114778WT) was isolated and sequenced by a Canadian research team (12). In contrast to the previously well-documented location of *grdA* on the Col-type plasmid pZJ18 (MT246861.1) (1, 2), our analysis revealed four strains harboring *grdA* on an IncHI2 plasmid, consistent with prior reports of this plasmid type in three *grdA*-positive isolates by Kim et al. (1). The remaining five strains had *grdA* integrated into their chromosome (Fig. S2). The genetic environment of each *grdA* forms a conserved unit originally characterized by Kim et al. (1), where *grdA* with IS*1394* (IS*30* family) immediately upstream is flanked by two copies of an IS*256* family transposase identified via ISEScan and ISFinder (IS*Maq7*-like; ISFinder BLASTn: 71.6% nt identity to IS*Maq7*) (13, 14), forming the structure IS*256-grdA*-IS*1394*-IS*256* (Fig. 1A and Fig. S3A). This 1,384 bp-long IS*256* family member had conserved terminal repeat sequences (IRs) of 25 bp (IRL: GAGTTATGGTGATTTCTGGTGTATG and IRR: GAGTTATTGTCAAATCTGGTGTATG) and generated 9 bp directly repeated duplication (DRs). This structure aligns with the canonical features of the IS256 family, which typically range from 1,200 to 1,500 bp, possess IRs of 20–30 bp, and generate DRs of 8–9 bp upon insertion (15). IS*256* family transposases, widely present in MDR staphylococci and enterococci, are frequently associated with the horizontal transfer of AMR genes (16, 17). In addition to sharing 99.7% sequence identity with part of Col-type plasmid pZJ18 (e.g., MT246861.1: 140-4869; Easyfig BLASTn: 99.7% nt identity to CP082667.1: 301082-305797), this composite transposon exhibits 100% sequence identity across chromosomal and plasmid contexts, suggesting a common IS*256* family-driven mobilization that enabled its spread between plasmids and chromosomes (Fig. 1B and Fig. S3B).

Seven strains carried one copy of *grdA* within IS*256-grdA*-IS*1394*-IS*256*, Albany CVM N18S2238 (CP049312.1) and Bredeney SA20114778WT (CP043222.1) carried two and three copies on their chromosomes, respectively (Fig. 1B). BLASTn analysis revealed that the structure of these tandem repeat regions shared 100.0% sequence identity with single-copy regions located on either chromosomes or plasmids (Fig. 1B), further indicating that the genetic context surrounding *grdA* remains conserved during transmission. The two adjacent repeat regions share a single IS*256*, suggesting that the tandem repeat structure of *grdA* is likely mediated by IS*256*. Interestingly, in contrast to the classic IS*256* compound transposon configuration (e.g., Tn*4001*, where resistance genes are flanked by inversely oriented IS*256* copies) (18, 19), the directly oriented IS*256* copies bracketing the *grdA*-IS*1394* unit in our identified basic structure are structurally analogous to the pseudo-compound transposons defined for IS26-family elements (20, 21). Furthermore, the observed multicopy arrays, characterized by shared central IS*256* copies, closely mirror the architecture of translocatable unit (TU) arrays described for IS*26* family mobilization. This structural congruence is indicative of analogous IS*256*-mediated mobilization mechanisms, involving TUs (such as IS*256-grdA*-IS*1394*) integrating adjacent to resident IS*256* copies via a pathway functionally similar to the targeted conservative cointegration implicated in IS*26*-family TU array formation (22). Nonetheless, the precise mechanistic details (whether aligning with traditional IS*256* transposition or IS*26*-like targeted events) remain to be subject to experimental verification. Furthermore, the presence of multiple copies of AMR genes is a potential contributor to increased antibiotic resistance (23, 24). In addition to chromosomal multicopy arrays, plasmid-mediated amplification (e.g., the Col-type plasmid) can also result in elevated levels of resistance (25, 26). Therefore, urgent attention is required to address the contribution of both multicopy genomic contexts, as they pose a significant challenge to the effectiveness of plazomicin.

Resistome profiling revealed that *grdA*-harboring strains consistently harbored several additional AMR genes, including those conferring resistance to aminoglycosides (*aac (3)-VIa*, *aac(6′)-Iaa*, *aac(6′)-Ib3*, *aadA1*, *aadA2*, *ant(2″)-Ia*, *aph(3″)-Ib*, and *aph (6)-Id*), beta-lactam (*bla*_CMY-2_ and *bla*_CTX-M-1_), fosfomycin (*fosA7*), sulfonamides (*sul1* and *sul2*), colistin (*mcr-9.1*), and tetracycline (*tet*(A), *tet*(B), and *tet*(C)) (Table 1, Fig. S1 and Table S3). These coexisting resistance genes indicate a complex resistance profile, posing challenges to the treatment of MDR bacterial infections. Notably, the presence of *mcr-9* alleles does not universally confer colistin resistance, as reported in the U.S. NARMS surveillance analysis by Tyson et al., which includes isolates co-harboring *grdA* and *mcr-9* from this study, such as CVM N18S1350, CVM N17S1352, CVM N53023, CVM N16S321, and CVM N58631 (27).

**TABLE 1:** Distribution and co-occurrence frequencies of AMR genes among *grdA*-positive *Salmonella enterica* strains in this study (*n* = 9)

Drug class	AMR gene	Positive strains	Number
Aminoglycoside	*aac (3)-VIa*	CVM N53023; CVM N16S321; CVM N58631	3
Aminoglycoside	*aac(6′)-Iaa^a^*	CVM 24358; SA20114778WT; CVM N18S0406; CVM N18S1350; CVM N18S2238; CVM N17S1352; CVM N53023; CVM N16S321; CVM N58631	9
Aminoglycoside	*aac(6′)-Ib3*	CVM 24358; CVM N18S1350; CVM N18S2238; CVM N53023; CVM N58631	5
Aminoglycoside	*aadA1*	CVM 24358; CVM N18S1350; CVM N18S2238; CVM N53023; CVM N16S321; CVM N58631	6
Aminoglycoside	*aadA2*	CVM 24358; SA20114778WT; CVM N18S0406	3
Aminoglycoside	*ant(2″)-Ia*	CVM 24358; SA20114778WT; CVM N18S0406	3
Aminoglycoside	*aph(3″)-Ib*	CVM 24358	1
Aminoglycoside	*aph (6)-Id*	CVM 24358	1
Beta-lactam	*bla*_CMY-2_	CVM 24358; CVM N18S0406	2
Beta-lactam	*bla*_CTX-M-1_	SA20114778WT; CVM N17S1352	2
Fosfomycin	*fosA7*	CVM N17S1352; CVM N53023; CVM N16S321; CVM N58631	4
Sulfonamide	*sul1*	CVM 24358; SA20114778WT; CVM N18S0406; CVM N53023; CVM N16S321; CVM N58631	6
Sulfonamide	*sul2*	CVM 24358	1
Colistin	*mcr-9.1*	CVM N18S1350; CVM N18S2238; CVM N17S1352; CVM N53023; CVM N16S321; CVM N58631	6
Tetracycline	*tet*(A)	CVM 24358	1
Tetracycline	*tet*(B)	CVM N18S1350; CVM N18S2238	2
Tetracycline	*tet*(C)	CVM 24358	1

^*a*^ Intrinsic AMR gene of *Salmonella*.

Our results provide additional insights into the IS*256*-driven mobilization of *grdA* between chromosomal and plasmid contexts, illustrating diverse trajectories of *grdA* spread among antibiotic-resistant *S. enterica*. By bridging chromosomal and plasmid dynamics and resistome profiling, we highlight the need to monitor potential resistance evolution through horizontal transfer and AMR gene amplification, which may impact next-generation antibiotics like plazomicin.

## SUPPLEMENTAL MATERIAL

Fig. S1 aac.00669-25-s0001.tif Core genome phylogeny of *S. enterica* serovars Bredeney, Albany, and Heidelberg.

Fig. S2 aac.00669-25-s0002.tif Circular representation of the chromosomes and plasmids of *S. enterica* strains harboring grdA.

Fig. S3 aac.00669-25-s0003.tif Clustal Omega sequence alignment.

Supplemental material aac.00669-25-s0004.docx Supplemental figure legends.

Supplemental tables aac.00669-25-s0005.docx Tables S1 and S2.

Table S3 aac.00669-25-s0006.xlsx Antimicrobial susceptibility profiles of *Salmonella enterica* by AMR phenotype prediction using ResFinder v4.3.1.

ASM does not own the copyrights to Supplemental Material that may be linked to, or accessed through, an article. The authors have granted ASM a non-exclusive, world-wide license to publish the Supplemental Material files. Please contact the corresponding author directly for reuse.
