# Supplementary figures and images for: grdA on different plasmids and chromosomes of Salmonella enterica
Source: Antimicrob Agents Chemother. 2025 Sep 22;69(11):e00669-25. doi: 10.1128/aac.00669-25 (PMC12587605; doi:10.1128/aac.00669-25)

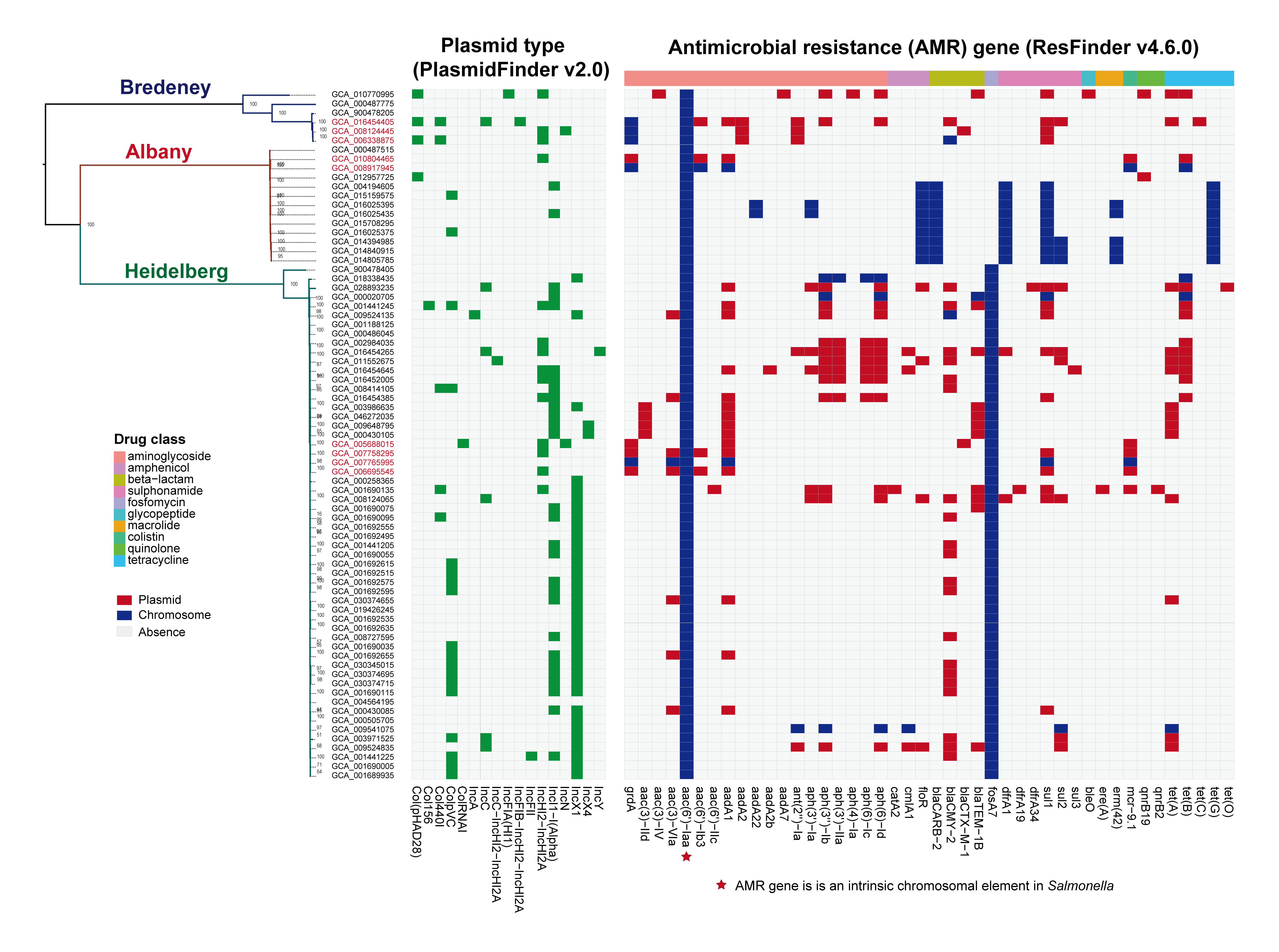

Supplement: Fig. S1 — Core genome phylogeny of S. enterica serovars Bredeney, Albany, and Heidelberg. [file aac.00669-25-s0001.tif]

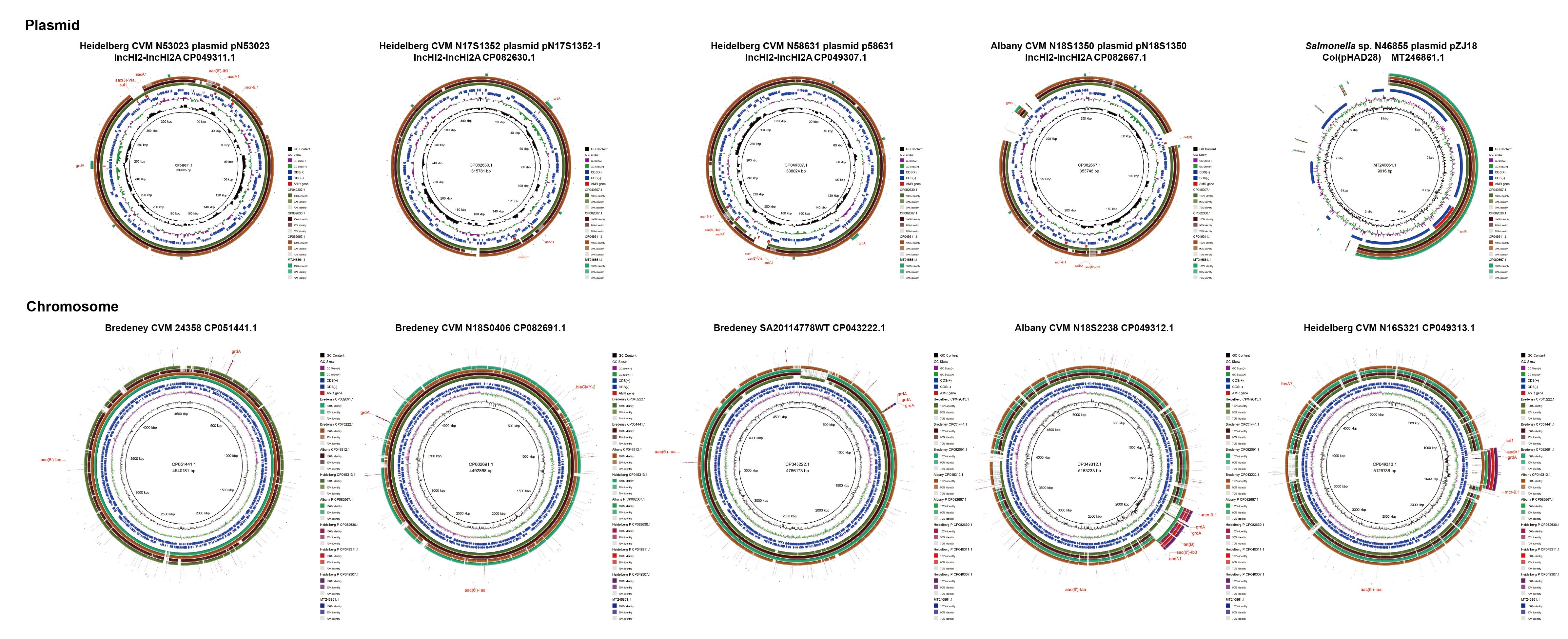

Supplement: Fig. S2 — Circular representation of the chromosomes and plasmids of S. enterica strains harboring grdA. [file aac.00669-25-s0002.tif]

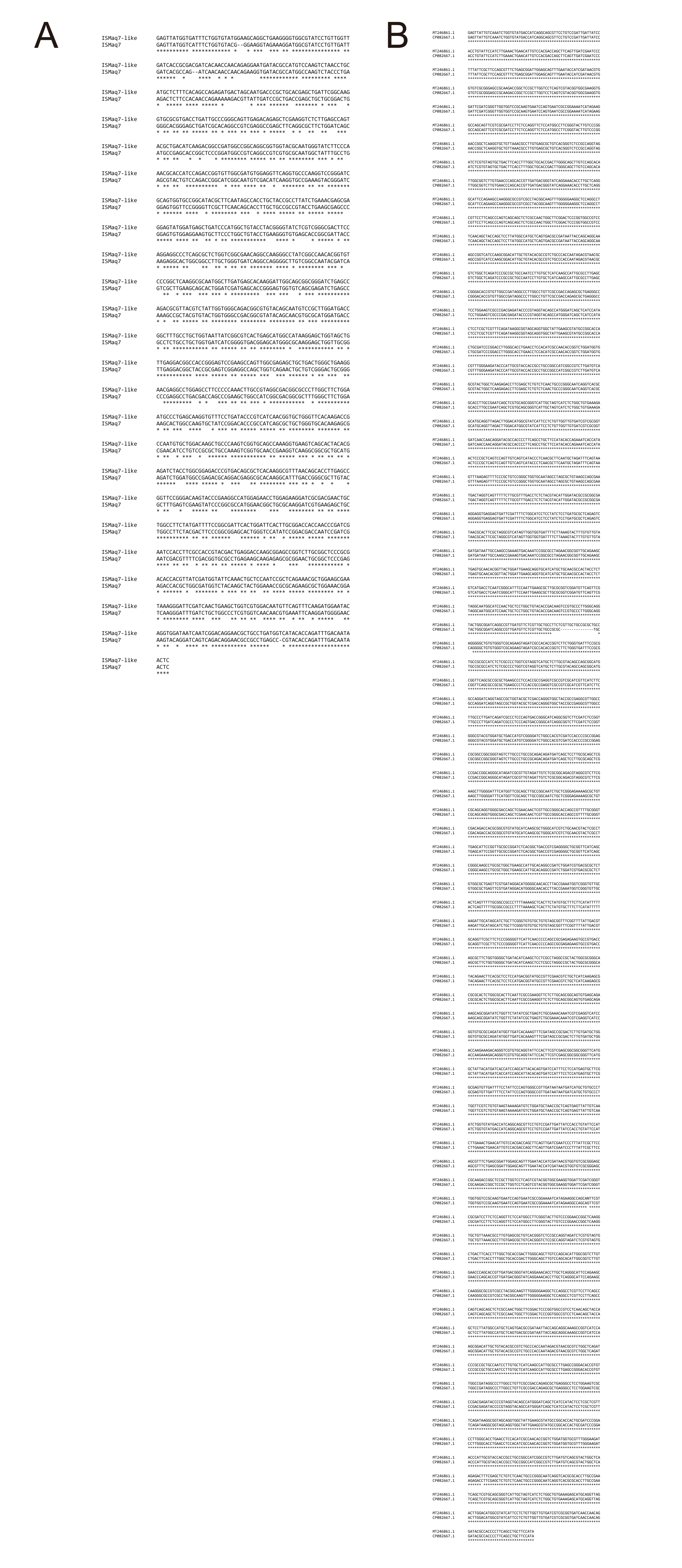

Supplement: Fig. S3 — Clustal Omega sequence alignment. [file aac.00669-25-s0003.tif]
