# Supplementary material for: grdA on different plasmids and chromosomes of Salmonella enterica
Source: Antimicrob Agents Chemother. 2025 Sep 22;69(11):e00669-25. doi: 10.1128/aac.00669-25 (PMC12587605; doi:10.1128/aac.00669-25)
Supplement: Supplemental material — Supplemental figure legends. [file aac.00669-25-s0004.docx]

**Supplemental Materials**

**Figure S1.** Core genome phylogeny of *S. enterica* serovars Bredeney, Albany, and Heidelberg. The maximum likelihood (ML) tree was constructed based on the core gene alignment shared by 75 *S. enterica* genomes. Adjacent to the tree are heatmaps showing plasmid types and the distribution of antimicrobial resistance (AMR) genes in the *S. enterica* genomes. The red asterisk indicates the intrinsic AMR gene of *Salmonella*.

**Figure S2.** Circular representation of the chromosomes and plasmids of *S. enterica* strains harboring *grdA*.

**Figure S3.** Clustal Omega sequence alignment. A. Sequence alignment of the IS*256* family transposase from pZJ18 (MT246861.1: 60 - 1443) with IS*Maq7*. B. Sequence alignment of the homologs (IS*256*-*grdA*-IS*1394*-IS*256*) between pZJ18 (MT246861.1: 140 – 4869) and pN18S1350 (CP082667.1: 301082 - 305797).
