# Supplementary material for: grdA on different plasmids and chromosomes of Salmonella enterica
Source: Antimicrob Agents Chemother. 2025 Sep 22;69(11):e00669-25. doi: 10.1128/aac.00669-25 (PMC12587605; doi:10.1128/aac.00669-25)
Supplement: Supplemental tables — Tables S1 and S2. [file aac.00669-25-s0005.docx]

Table S1. Public (complete) genome sequences containing grdA identified using the NCBI BLASTn (accessed on 9 April 2025) against the nucleotide collection (nr/nt) database.

| **Serovar** | **Strain name** | **Isolation source** | **Geographic location** | **Collection date** | **grdA location** | | | **BioProject** | | **Publication** |
| --- | --- | --- | --- | --- | --- | --- | --- | --- | --- | --- |
|  |  |  |  |  | **Location** | **Accession** | **Position in contig** |  |  |  |
| Heidelberg | CVM N16S321 | ground turkey | USA:NY | 2016 | chromosome | CP049313.1 | 1218277...1218783 | PRJNA292661 | Whole genome sequencing of foodborne pathogens as part of the US Food and Drug Administration surveillance project for NARMS. | Tate *et al*. A National Antimicrobial Resistance Monitoring System Survey of Antimicrobial-Resistant Foodborne Bacteria Isolated from Retail Veal in the United States. J Food Prot. 2021 Oct 1;84(10):1749-1759. |
| Heidelberg | CVM N17S1352 | ground turkey | USA:MD | 2017 | plasmid | CP082630.1 | 41029...41535 | PRJNA292661 |  |  |
| Heidelberg | CVM N58631 | ground turkey | USA:CT | 2015 | plasmid | CP049307.1 | 131833...132339 | PRJNA292661 |  |  |
| Heidelberg | CVM N53023 | ground turkey | USA:GA | 2014 | plasmid | CP049311.1 | 254767...255273 | PRJNA292661 |  |  |
| Albany | CVM N18S2238 | ground turkey | USA:CO | 2018 | chromosome | CP049312.1 | 1744318...1744824; 1747830...1748336 | PRJNA292661 |  |  |
| Albany | CVM N18S1350 | ground turkey | USA:TN | 2018 | plasmid | CP082667.1 | 303951...304457 | PRJNA292661 |  |  |
| Bredeney | CVM N18S0406 | ground turkey | USA:MD | 2018 | chromosome | CP082691.1 | 3685180...3685686 | PRJNA292661 |  |  |
| Bredeney | SA20114778WT | turkey | - | 2016 | chromosome | CP043222.1 | 761904...762410; 765416...765922; 768928...769434 | PRJNA560883 |  |  |
| Bredeney | CVM 24358 | turkey | USA:MO | 2002 | chromosome | CP051441.1 | 314375...314881 | PRJNA622256 |  |  |
| Heidelberg | CVM N16S321 | ground turkey | USA:NY | 2016 | chromosome | CP049313.1 | 1218277...1218783 | PRJNA292661 |  |  |
| Heidelberg | CVM N17S1352 | ground turkey | USA:MD | 2017 | plasmid | CP082630.1 | 41029...41535 | PRJNA292661 |  |  |
| Heidelberg | CVM N58631 | ground turkey | USA:CT | 2015 | plasmid | CP049307.1 | 131833...132339 | PRJNA292661 | Inflammatory infection and transmission of β-lactam resistance in a mouse model of ampicillin-induced microbiota | Laskey *et al*. Mobility of β-Lactam Resistance Under Bacterial Co-infection and Ampicillin Treatment in a Mouse Model. Front Microbiol. 2020 Jul 7;11:1591. |
| Heidelberg | CVM N53023 | ground turkey | USA:GA | 2014 | plasmid | CP049311.1 | 254767...255273 | PRJNA292661 | Comparative Genomic Analysis of *Salmonella* Strains Isolated from Diseased Animals. | Zhao *et al*. Comparative Genomic Analysis of 450 Strains of *Salmonella enterica* Isolated from Diseased Animals. Genes (Basel). 2020 Sep 1;11(9):1025. |

Table S2. *grdA*-positive *Salmonella* isolates from prior genomic study (Kim *et al*. 2020, doi: 10.1128/AAC.00867-20.).

| **Assembly** | **Subspecies** | **Serovar** | **Strain name** | **Isolation source** | **Geographic location** | **Collection date** |
| --- | --- | --- | --- | --- | --- | --- |
| GCA_001478645.1 | *Salmonella enterica* subsp. *enterica* | Bredeney | CVM N29351 | Ground Turkey | USA: TN | 2011 |
| GCA_001479265.1 | *Salmonella enterica* subsp. *enterica* | Heidelberg | CVM N31844 | Ground Turkey | USA: MD | 2011 |
| GCA_001479465.1 | *Salmonella enterica* subsp. *enterica* | Senftenberg | CVM N32755 | Ground Turkey | USA: MN | 2011 |
| GCA_001479525.1 | *Salmonella enterica* subsp. *enterica* | Senftenberg | CVM N32779 | Ground Turkey | USA: NM | 2011 |
| GCA_001243145.1 | *Salmonella enterica* subsp. *enterica* | Albany | CVM N46827 | Ground Turkey | USA: GA | 2013 |
| GCA_001243615.1 | *Salmonella enterica* subsp. *enterica* | Muenchen | CVM N46855 | Ground Turkey | USA: PA | 2013 |
| GCA_001272375.1 | *Salmonella enterica* subsp. *enterica* | Heidelberg | CVM N51271 | Ground Turkey | USA: MD | 2013 |
| GCA_001247075.1 | *Salmonella enterica* subsp. *enterica* | Heidelberg | CVM N51292 | Ground Turkey | USA: NM | 2013 |
